# Severe cutaneous allergic reactions in Brazil: new risk alleles to be identified in our population?

**DOI:** 10.1186/1939-4551-8-S1-A107

**Published:** 2015-04-08

**Authors:** Maria Ines Perelló, Camila M Filgueiras, Fernanda Marques Conceição, Natalia Rocha Estanislau, Denise Lacerda Pedrazzi, AssunÇao De Maria Gusmao Ferreira Castro, Sonia Conte Caracciolo Costa, Eduardo Costafsilva

**Affiliations:** 1 State University of Rio De Janeiro, Brazil; 2 Instituto De Dermatologia PROF Rubem David Azulay, Brazil; 3 Brazillian Association of Allergy and Immunology, Brazil

## Background

Severe cutaneous allergic reactions (SCAR) have a wide range of severity and clinical manifestations. Some risk alleles are known in specific populations. The aim of the study was to describe the clinical and laboratory profile of patients with SCAR, treated at a university hospital in Rio de Janeiro.

## Methods

This is a retrospective cross-sectional study. Clinical and laboratory data, including HLA typing, of patients with SCAR identified between March/2011 and July/2014 by pharmacologic surveillance, weekly done by our Service, were reviewed.

## Results

Twenty-three cases of SCAR were identified: 12 DRESS/DIHS, 1 overlap DRESS/AGEP and 10 SJS/TEN. Sixteen patients (70%) were female, the median age was 41 years (IQR=26-50). The aromatic anticonvulsivants were implicated in the etiology in 73% of cases, followed by antibiotics (30%). All patients with DRESS/DIHS exhibited cutaneous, systemic and laboratory characteristic changes of this syndrome. Patients with SJS/TEN had fever and mucosal involvement, 20% had neurological abnormalities and no one organ involvement or ocular complications. Eight (66%) patients with DRESS had late reactivation of disease. There was 1 death due to refractory cardiac insufficiency. During the 1^st^year of outpatient follow-up, we found autoimmune changes in 21% of patients. Reactions to others drugs following diagnosis occurred in 2 patients with DRESS/DIHS, 1 patient with SJS/TEN had solar urticaria and another one with DRESS/DIHS developed dermographism. All patients with DRESS/DIHS were treated with corticosteroids, with an average of 108 days (9-180) of treatment. Eight out of 10 patients with SJS/TEN used corticosteroids with good response. IGIV was used in 1 patient with SJS/TEN and associated with steroids in 1 patient with DRESS/DIHS. We identified the known relationship between carbamazepine (CBZ) and HLA-A*31:01 in 2 patients with DRESS and allopurinol and HLA-B*58:01 in 2 patients (one SJS/TEN and one overlap DRESS/AGEP). Interestingly, we found the same alleles in 3 patients with DRESS caused by phenytoin (HLA-A*23 and HLA-B*53) and in 3 (DRESS) with carbamazepine (HLA-A*74 and HLA-B*15). None of them were family related.

## Conclusions

This study confirmed the main clinical and laboratory features of SCAR. The correct and early diagnosis of these reactions allowed the effective management and ambulatory monitoring, with a good outcome in most cases. The HLA typing can corroborate the diagnosis in 4 cases. The repetition of still not described alleles in our patients deserves larger studies to identify if these alleles are associated with higher risk of SCAR in our population.

